# Specificity, redundancy and dosage thresholds among *gata4/5/6* genes during zebrafish cardiogenesis

**DOI:** 10.1242/bio.053611

**Published:** 2020-06-24

**Authors:** Jessica Sam, Emily J. Mercer, Ingrid Torregroza, Kelly M. Banks, Todd Evans

**Affiliations:** Department of Surgery, Weill Cornell Medicine, New York, NY 10065, USA

**Keywords:** Embryogenesis, Heart development, Morphogenesis, Progenitor cells, Transcriptional regulation

## Abstract

The Gata4/5/6 sub-family of zinc finger transcription factors regulate many aspects of cardiogenesis. However, critical roles in extra-embryonic endoderm also challenge comprehensive analysis during early mouse cardiogenesis, while zebrafish models have previously relied on knockdown assays. We generated targeted deletions to disrupt each *gata4/5/6* gene in zebrafish and analyzed cardiac phenotypes in single, double and triple mutants. The analysis confirmed that loss of *gata5* causes *cardia bifida* and validated functional redundancies for *gata5/6* in cardiac precursor specification. Surprisingly, we discovered that *gata4* is dispensable for early zebrafish development, while loss of one *gata4* allele can suppress the *bifid* phenotype of the *gata5* mutant. The *gata4* mutants eventually develop an age-dependent cardiomyopathy. By combining combinations of mutant alleles, we show that cardiac specification depends primarily on an overall dosage of *gata4/5/6* alleles rather than a specific gene. We also identify a specific role for *gata6* in controlling ventricle morphogenesis through regulation of both the first and second heart field, while loss of both *gata4/6* eliminates the ventricle. Thus, different developmental programs are dependent on total dosage, certain pairs, or specific *gata4/5/6* genes during embryonic cardiogenesis.

This article has an associated First Person interview with the first author of the paper.

## Abstract (precis)

**Summary:** Targeted mutations were generated for each of the three *gata4/5/6* genes in zebrafish to define functions for individual or combinations of these related transcription factors during cardiogenesis.

## INTRODUCTION

The GATA family of zinc finger transcription factors are key drivers of gene regulatory networks for many organ systems during embryonic development. GATA1-6 are highly conserved across vertebrate species, with GATA4/5/6 directing development of mesendoderm and subsequently cardiac mesoderm and endoderm-derived organs (Tremblay et al., 2018). Mutations in each of these three genes are linked to rare haplo-insufficient congenital heart defects in humans, while disruption of each gene causes a variety of abnormal heart morphogenesis and maturation phenotypes in animal models (Wolf and Basson, 2010; McCulley and Black, 2012; Shan et al., 2014). Despite the clear importance of GATA factors in directing heart development, elucidating each of their specific and combinatorial roles has been challenging for several reasons. The genes have largely overlapping expression patterns that may facilitate genetic compensation. Both *GATA4* and *GATA6* are essential for mammalian extra-embryonic endoderm development (Molkentin et al., 1997; Zhao et al., 2008), necessitating conditional alleles or tetraploid complementation assays to probe their function in cardiogenesis. Other animal models including *Xenopus* and zebrafish have lacked null alleles and relied mostly on knockdown assays, in particular using morpholinos.

Despite these challenges, studies analyzing knockout or knockdown for GATA4/5/6 in animal models have revealed a range of cardiac requirements. Using a ‘cardiac specific’ *Nkx2.5*:Cre driver, loss of murine Gata4 caused defects in cardiomyocyte proliferation and right ventricle morphogenesis (Zeisberg et al., 2005), while loss of murine Gata6 caused ventricular septal and trabeculation defects (van Berlo et al., 2010). However, the genes are also associated with the development of earlier mesendoderm stages, and likely function upstream of *Nkx2.5* (Lien et al., 1999) so these experiments may under-estimate the full requirement for cardiogenesis. Murine tetraploid complementation studies suggest that Gata4 is required not in embryonic myocardium but for formation of epicardium, and its loss leads indirectly to cardiac morphogenetic defects (Watt et al., 2004). Similar experiments indicated that Gata6 is not required embryonically for cardiogenesis (Zhao et al., 2005). This is somewhat surprising, since conditional knockouts in neonates indicate functions in neonatal cardiomyocytes for growth (Prendiville et al., 2015) and in adults for hypertrophic response (van Berlo et al., 2010). One explanation could be redundancies in GATA factor function, as demonstrated previously for cardiac specification by *gata5/6* using morpholinos in zebrafish (Holtzinger and Evans, 2007), and for *GATA4/6* by tetraploid complementation in mice (Zhao et al., 2008). In addition, *GATA4/5* double mutant mice display chamber septal defects and abnormal ventricular development (Xin et al., 2006; Singh et al., 2010).

Since zebrafish embryos do not rely on the equivalence of mammalian extra-embryonic endoderm for early development, they provide an excellent model to probe genetic interactions among these genes during cardiogenesis. The zebrafish *faust* mutant displays *cardia bifida*, due to the failure of progenitors to form a midline primitive heart tube. The *fau^s26^* allele was mapped to *gata5* (Reiter et al., 1999), but the genetic lesion associated with the mutant remains unknown. In addition, no studies have reported the impact of triple *Gata4/5/6* knockouts in any animal model. Therefore, we generated mutant zebrafish lines harboring targeted deletions in the essential C-terminal DNA binding domain of *gata4/5/6*. In addition, we out-crossed single mutant lines to generate multiple combinations of double and triple *gata4/5/6* heterozygous and homozygous mutant offspring to define the individual and redundant functions of *gata4/5/6* during different stages of embryonic heart development. In zebrafish, *gata4* is not required for embryonic cardiogenesis. The role of *gata5* in heart tube formation was confirmed, while *gata6* was found to be critical for ventricle development. By evaluating embryos with defined numbers of mutant alleles, we describe evidence for genetic compensation and dosage requirements for Gata4/5/6-dependent cardiogenesis.

## RESULTS

### Gata4 is dispensable for early zebrafish development

**Fig. 1.**
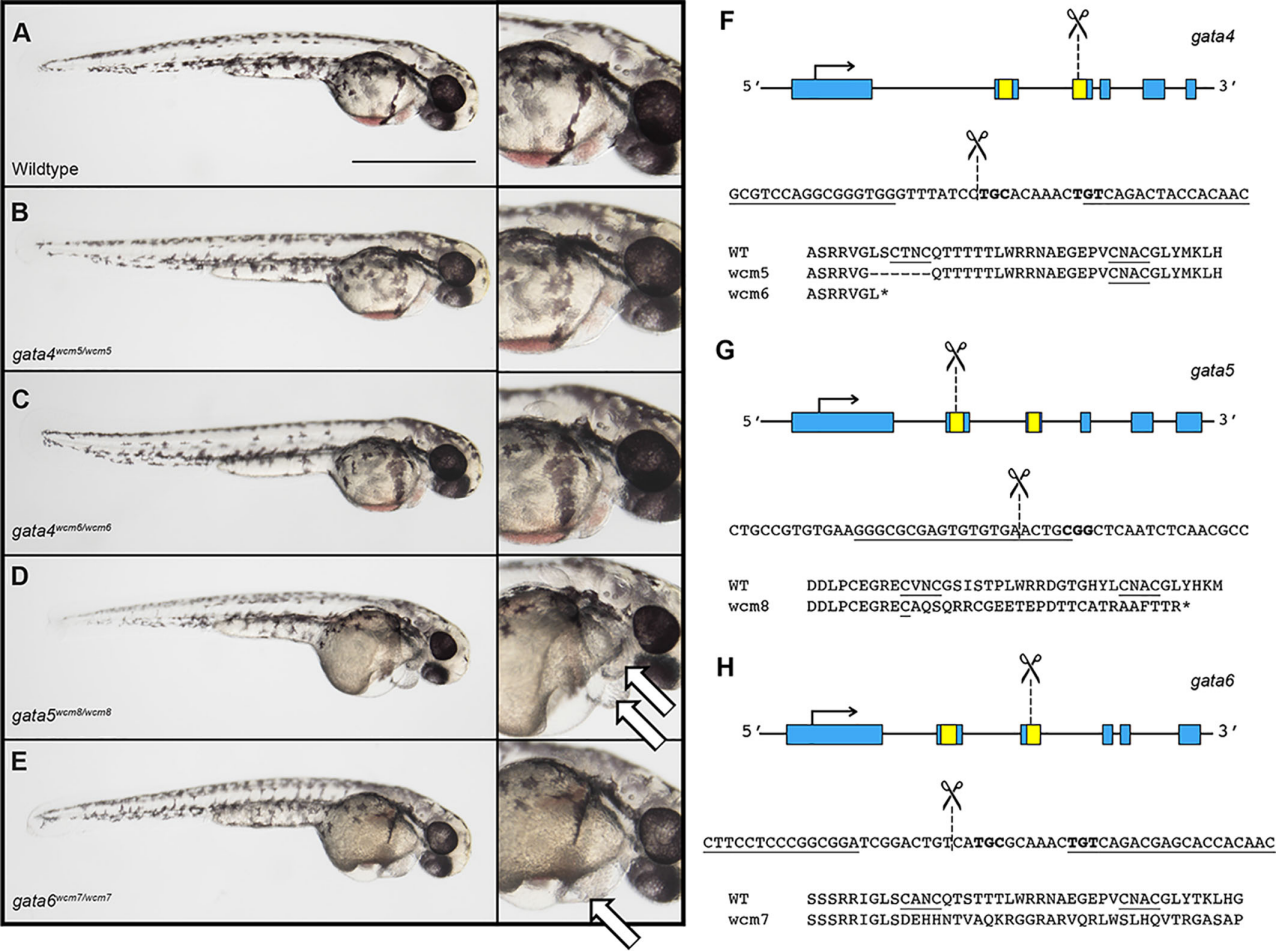
**Mutation of *gata4* is tolerated while mutations** **in *gata5* or *gata6* cause embryonic lethal cardiac defects.** (A–E) Representative images of 2 dpf *gata4*, *gata5*, or *gata6* homozygous mutants compared to wild type following TALEN (*gata4* and *gata6*) or CRISPR (*gata5*) mediated deletion of the GATA zinc finger DNA-binding domain. (B,C) *Gata4* homozygous mutants are phenotypically normal. (D,E) *Gata5* and *gata6* homozygous mutants develop severe pericardial edemas (indicated by white arrows in the far right panels) and fail to survive beyond 5–6 days. These phenotypes are 100% penetrant. Representative scale bar in A: 0.5 mm. (F–H) Schematic showing the structure, partial nucleotide sequence, and partial amino acid sequence for each of the *gata4/5/6* mutant alleles. Zinc finger domains are shown in yellow and regions targeted for deletion are marked by dashed lines.

Previous studies demonstrated that the C-terminal zinc finger encodes a DNA-binding domain that is essential for GATA factor function (Trainor et al., 1996); mutation of a single cysteine residue is sufficient to completely eliminate capacity for transcription factor activity (Yang and Evans, 1992). Therefore, we used TALEN and CRISPR technology to create targeted deletions of the C-terminal DNA binding domain in *gata4*, *gata5* or *gata6* (Table S1). For each gene, we recovered at least one mutant allele harboring a deletion of an essential C-X-X-C motif, and also resulting in a frame shift that introduced an early stop codon predicted to produce a truncated and functionally dead protein (Table S2). Using these lines, we generated single, double and triple heterozygous *gata4/5/6* zebrafish lines*.* Homozygous mutant embryos obtained from crosses of either *gata5* or *gata6* heterozygous adults developed pericardial edema by 2 days post-fertilization (dpf) that subsequently resulted in embryonic lethality (Fig. 1D,E, white arrows). In addition, *gata5^−/−^* embryos developed *cardia bifida*, a phenotype described previously in *gata5* morphants and the *gata5 faust* mutant (Fig. S1; Reiter et al., 1999; Trinh et al., 2005; Holtzinger and Evans, 2007). Importantly, the generation of a defined *gata5* mutant facilitates genotyping required to identify new combinations of mutants.

**Fig. 2.**
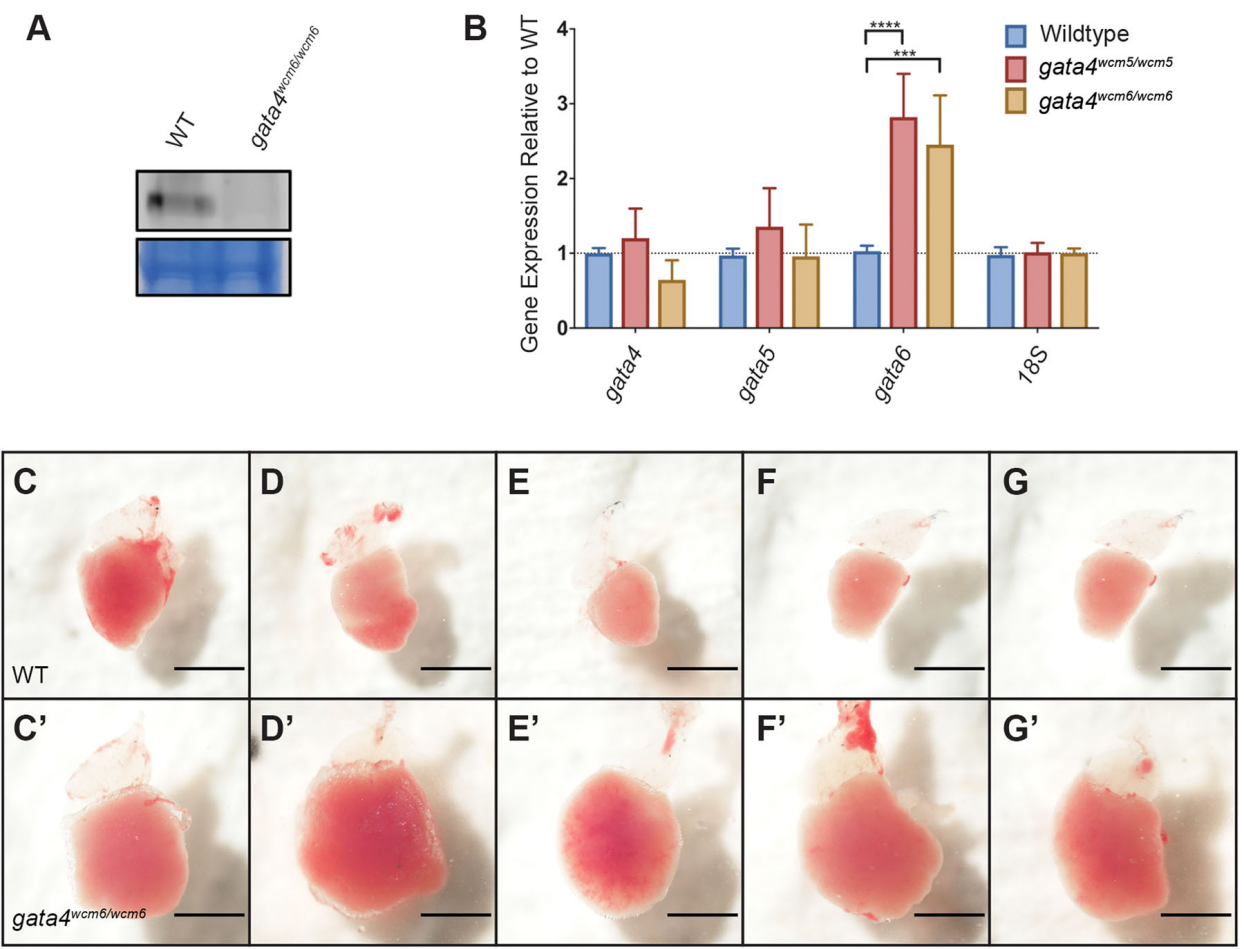
**The *gata4* mutants are viable to adulthood, but develop an age-dependent cardiomyopathy.** (A) Representative western blot showing the absence of Gata4 protein in dissected adult *gata4^−/−^* heart tissue. (B) Quantification by qPCR of *gata4/5/6* expression reveals a significant upregulation of *gata6* mRNA in *gata4^−/−^* embryos at the 50% epiboly stage. (C–G) Ventricles dissected from adult wild-type fish, representing phenotypically normal hearts. (C′–G′) Similarly dissected hearts from 10-month-old *gata4^−/−^* adults show severe cardiomyopathy and an enlarged ventricular chamber. Scale bar: 1 mm.

Strikingly, Gata4 is not required for zebrafish embryonic development, as *gata4^−/−^* mutant embryos obtained from parents heterozygous for either of two independently isolated indel alleles appeared phenotypically normal (Fig. 1B,C). We note that *gata4* null mutants were recovered at below the expected 25% ratio at both the embryonic and adult stages. Although the *gata4* mutant embryos were not found at Mendelian ratios, most of the embryos that were recovered survived to adulthood without exhibiting any obvious abnormalities (Fig. S2). To confirm that the *gata4* mutation is null, the absence of Gata4 protein in *gata4^−/−^* adult hearts was verified by western blotting (Fig. 2A). However, on close inspection, the *gata4* mutation does cause an embryonic phenotype, as *gata4^−/−^* embryos showed delayed gastrulation beginning at the 50% epiboly stage, after which they resumed development at a normal rate (Fig. S3). Previous studies have shown upregulation of *Gata6* in *Gata4* mutant mouse embryos (Molkentin et al., 1997) and of *Gata4* and *Gata6* in the hearts of *Gata5* null mice (Singh et al., 2010), suggesting that increased expression of at least one GATA factor may compensate for the loss of another. To determine if this occurs in the *gata4* mutant zebrafish embryos, qPCR experiments were performed to measure the relative expression level of each gene in *gata4* null embryos. Indeed, compared to wild-type embryos, the mutant embryos showed elevated levels of *gata6* mRNA, but not *gata4* or *gata5* mRNA (Fig. 2B). *Gata4* maternal-zygotic (m/z) mutants were raised to adulthood and appear phenotypically normal.

Although embryos can tolerate loss of *gata4*, the mutation eventually leads to cardiomyopathy. Strikingly, dissection of *gata4^−/−^* adult hearts at 10 months revealed gross abnormalities, including a significantly enlarged ventricle, compared to hearts from wild-type animals (Fig. 2C–G′). These defects were not observed in younger 3-month-old fish, indicating that *gata4* mutants recover from a delay in gastrulation to initially appear normal, but they gradually develop cardiomyopathy with age.

### The *gata4/5/6* genes exhibit functional redundancy for cardiac specification and heart tube morphogenesis

**Fig. 3.**
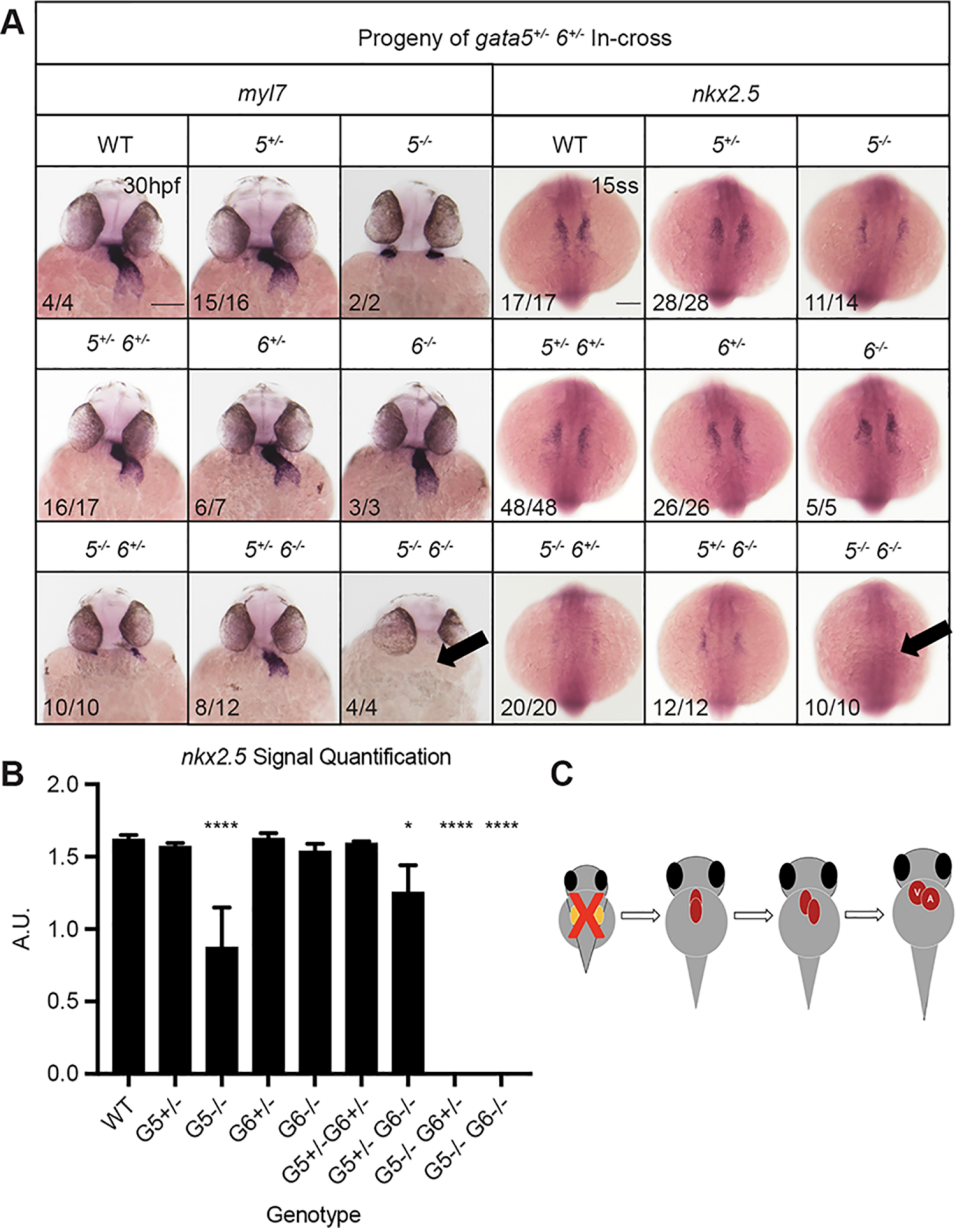
***Gata5* and *gata6* are redundant for cardiac specification.** ISH was performed using offspring from a *gata5^+/−^, gata6^+/−^* in-cross with probes targeting either myocardial marker *myl7* (left panels) or cardiac progenitor marker *nkx2.5* (right panels). (A) *gata5^−/−^, gata6^−/−^* mutants exhibit a heartless phenotype and a complete loss of cardiac progenitors (indicated by arrows). Representative scale bar in WT panels: 0.1 mm. (B) Quantification of *nkx2.5* staining shown in A. Error bars represent s.e.m. *P<0.05, ****P<0.0001. (C) Schematic showing that loss of *gata5* and *6* disrupts the initial stages of heart development.

We next compared the phenotypes of single and double mutants. To assess defects in heart tube morphogenesis, *in situ* hybridization (ISH) was performed using the cardiac-specific *myl7* probe with embryos collected from crosses of double heterozygous *gata5^+/−^;6^+/−^*, *gata4^+/−^;5^+/−^*, or *gata4^+/−^;6^+/−^* adults. For the *gata5^+/−^;6^+/−^* in-crosses, at 30 hpf bilateral fields of *myl7* expressing cardiomyocytes were observed in embryos harboring *gata5^−/−^* alleles, consistent with the *cardia bifida* phenotype described above and in *gata5* morphants (Trinh et al., 2005; Holtzinger and Evans, 2007) (Fig. 3A, left panels). The *gata5/6* homozygous mutants lacked cardiomyocytes, with the reduction of *myl7* staining becoming increasingly more severe with the progressive loss of *gata5/6* alleles. This is consistent with our previous studies using morpholino knockdowns that demonstrated functional redundancy between *gata5* and *gata6* for cardiac progenitor specification (Holtzinger and Evans, 2007), but also suggests a dosage affect for *gata5/6*, since the *gata5^−/−^;gata6^+/−^* embryo defect is more severe than seen in *gata5^−/−^; gata6^+/+^* embryos. To confirm that the lack of a heart tube in the genetic mutants is due to a loss of cardiac precursors, ISH experiments were performed with embryos at the 15-somite stage using a probe for the cardiac progenitor marker *nkx2.5*. As predicted, ISH revealed a complete loss of cardiac specification in *gata5/6* double null embryos (Fig. 3A, right panels). Quantification of the *nkx2.5* staining (Fig. S4) showed a much less severe but still significant reduction of *nkx2.5+* cells in the homozygous *gata5* mutant embryos, while loss of even one *gata6* allele essentially eliminates cardiac specification (Fig. 3B,C).

**Fig. 4.**
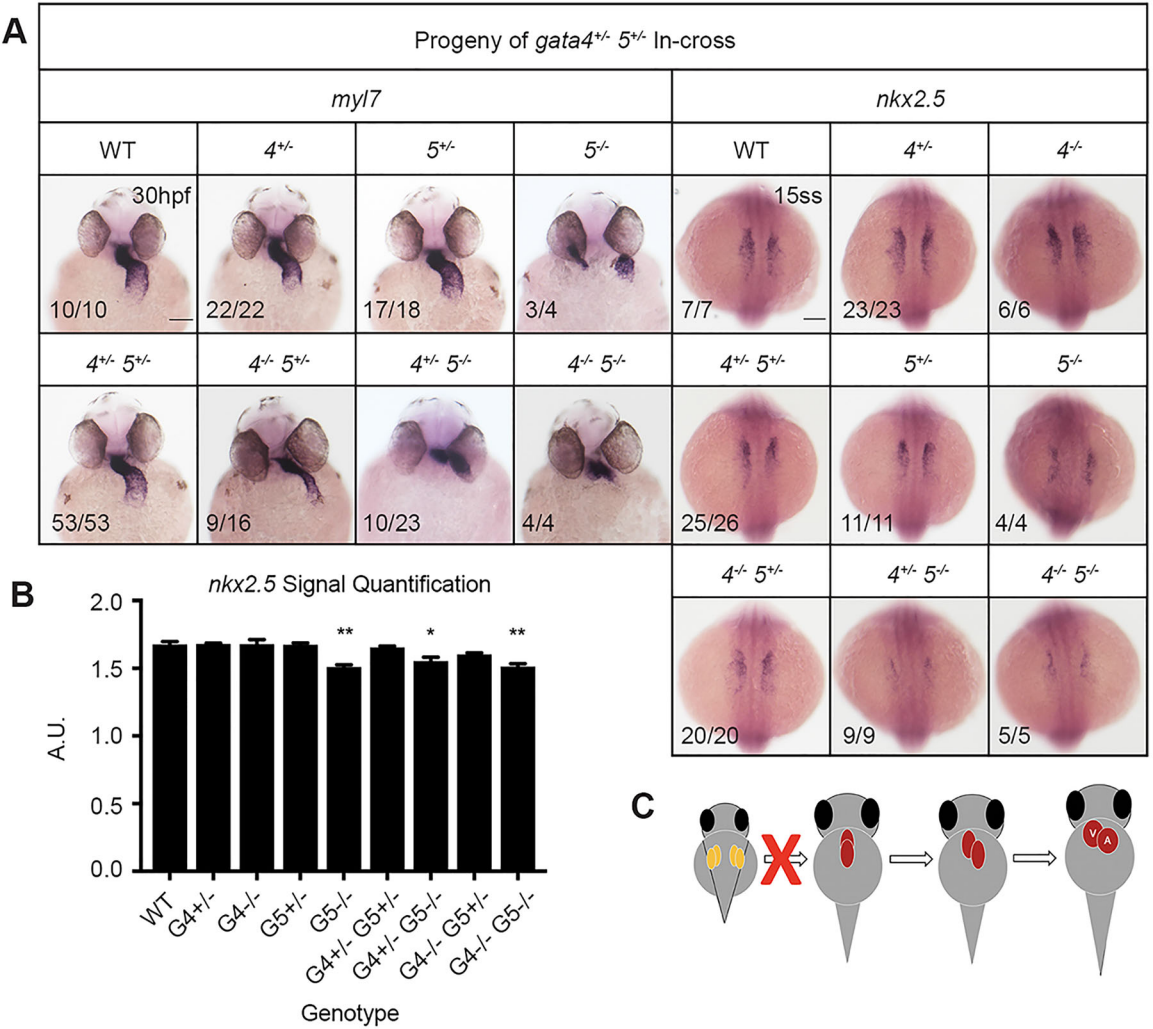
**Genetic interactions between gata4 and gata5 impact progenitor migration and heart tube formation.** ISH was performed using offspring from a *gata4^+/−^, gata5^+/−^* in-cross and probes targeting either myocardial marker *myl7* or cardiac progenitor marker *nkx2.5*. (A) Progressive loss of *gata4* and *gata5* alleles results in a range of heart tube defects due to abnormal heart tube morphogenesis. Loss of a gata5 allele in gata4 mutants generates a thin heart tube, while loss of *gata4* alleles partially rescues the *gata5^−/−^ bifid* phenotype. Representative scale bars in WT panels: 0.1 mm. (B) Quantification of *nkx2.5* staining shown in A shows that loss of *gata4* does not significantly affect cardiac specification. Error bars shown are s.e.m. *P<0.05, **P<0.01. (C) Schematic showing combinatorial loss of *gata4* and *gata5* disrupts proper fusion of the heart tube.

Embryos obtained by crossing *gata4/5* double heterozygous adults showed normal heart tube formation when only mutant for *gata4* and the expected *bifid* phenotype when only mutant for *gata5* (Fig. 4A). However, many of the *gata4* mutants carrying one mutant *gata5* allele displayed unusually thin heart tubes. Furthermore, the loss of one or two *gata4* alleles in the context of *gata5* null embryos partially rescued *cardia bifida*; in the double mutants, cardiac tube tissue was found at the midline, although incomplete fusion resulted in a severely dysmorphic heart tube (Fig. 4A, left panels). Both results suggest genetic interactions between *gata4* and *gata5*. Quantification of *nkx2.5* signals indicate that only embryos lacking both *gata5* alleles show a minor defect in specification (Fig. 4B), suggesting that interaction with *gata4* is at a later stage of morphogenesis (Fig. 4C).

**Fig. 5.**
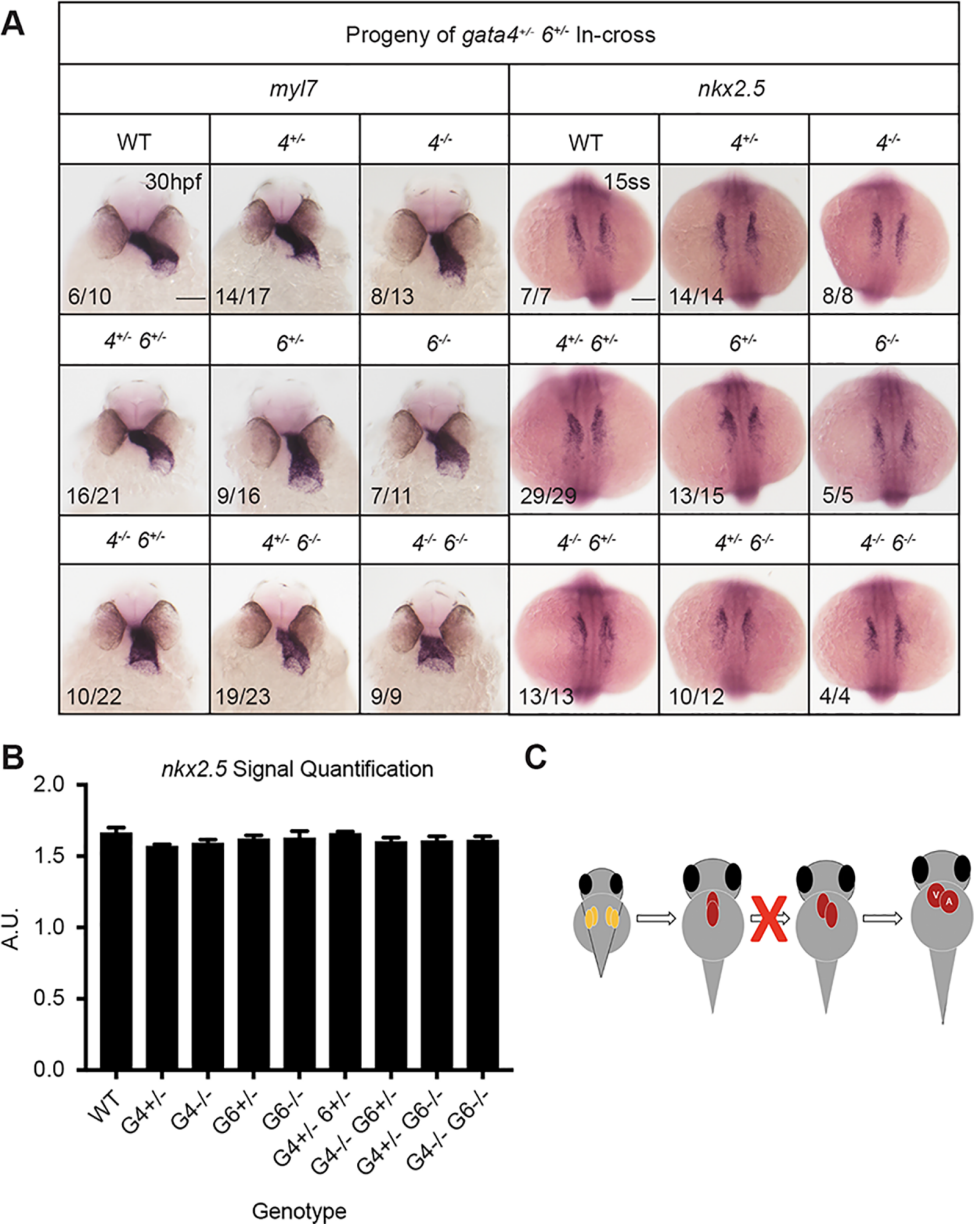
**Loss of *gata4* and *gata6* results in heart tube morphogenesis defects.** ISH was performed using offspring from a *gata4^+/−^, gata6^+/−^* in-cross and probes targeting either myocardial marker *myl7* or cardiac progenitor marker *nkx2.5*. (A) The *gata4* and *gata6* genes are partially redundant for heart tube elongation and looping. The *gata4^−/−^, gata6^−/−^* mutants display distinctly truncated and linear heart tubes. Representative scale bars in WT panels: 0.1 mm. (B) Loss of *gata4* and/or *gata6* does not affect cardiac specification. Error bars shown are s.e.m. (C) Schematic showing that combinatorial loss of *gata4* and *gata6* impairs the heart tube elongation stage of cardiogenesis.

By combining mutant alleles, functional redundancy was also observed for *gata4* and *gata6* with respect to heart tube morphogenesis. Abnormal heart tube looping (relatively short, linear heart tubes) was observed in both *gata4* and *gata6* single mutant embryos derived from crossing gata4/6 heterozygous adults, based on expression pattern by ISH for *myl7*, although this phenotype was not fully penetrant (Fig. 5A, left panels). However, the looping and short tube phenotype increased in severity and demonstrated full penetrance in *gata4/6* double mutants. Additional ISH experiments indicated there was no significant loss of cardiac precursors in either single or double mutants compared to wild-type siblings, suggesting that the phenotypes are not due to defects in specification of cardiac progenitors (Fig. 5A right panels, 5B). Since only the double mutant embryos exhibit distinctly linear, truncated heart tubes, the data suggest functional redundancy between *gata4* and *gata6* during heart tube elongation (Fig. 5C).

### Dosage of *gata4/5/6* alleles regulates cardiac cell fate

**Fig. 6.**
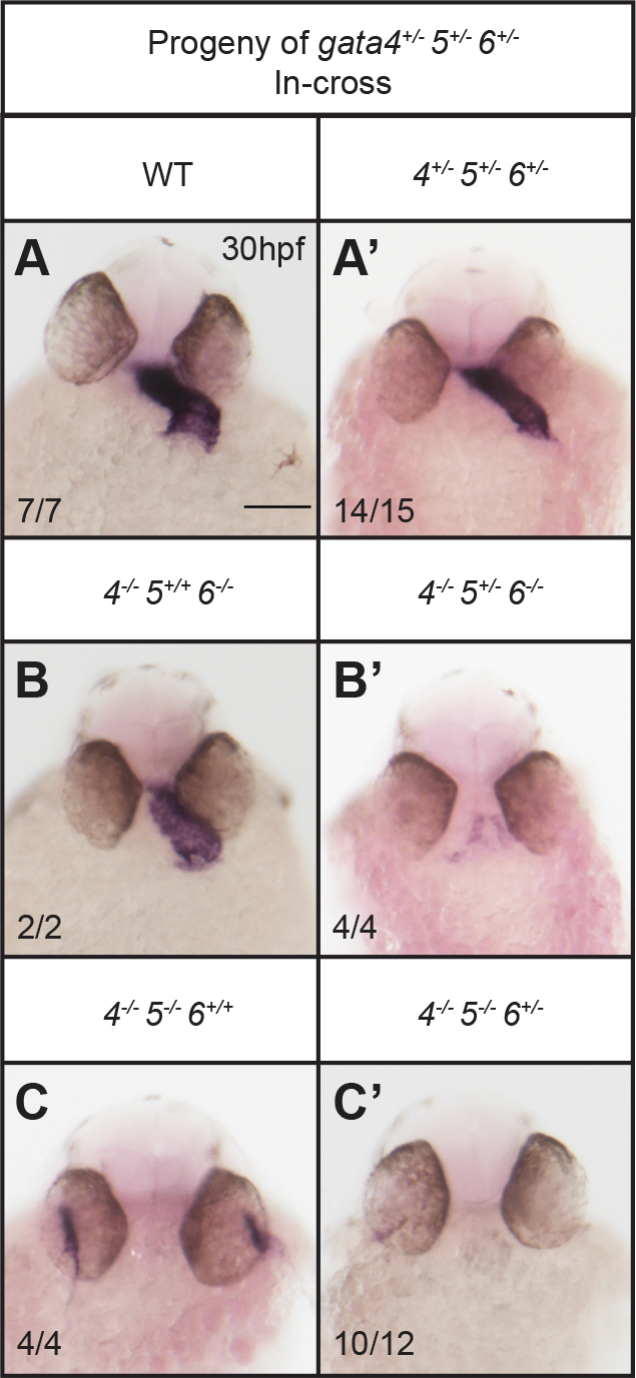
**Cardiac development depends on the dosage of *gata4/5/6* alleles.** ISH was performed using offspring from crossing *gata4^+/−^, gata5^+/−^, gata6^+/−^* triple heterozygous adults and a probe targeting myocardial marker *myl7*. (A,A′) Triple heterozygous embryos display abnormal heart tube morphology compared to wild-type siblings, although they can survive to adulthood. Loss of a single *gata5* or *gata6* allele in a *gata4^−/−^, gata6^−/−^* (B,B′) or *gata4^−/−^, gata5^−/−^* (C,C′) background, respectively, results in a severe loss of cardiac tissue. Representative scale bar in WT panel: 0.1 mm.

Some previous studies using mutant mouse models suggested that cardiac development is affected by the overall expression levels of GATA factors (Pu et al., 2004; Xin et al., 2006). To more thoroughly investigate this concept, we varied the allele frequency for all three *gata4/5/6* genes by crossing *gata4/5/6* triple heterozygous adults and analyzing the offspring for cardiac phenotypes based on the ISH expression pattern of *myl7*. Triple heterozygous offspring have relatively thin and straight heart tubes compared to wild-type siblings at 30 hpf (Fig. 6A,A′), yet these fish subsequently develop normally and are viable to adulthood. In contrast, embryos harboring homozygous null mutations for at least two *gata4/5/6* genes suffered a severe loss of cardiac tissue with the additional loss of a fifth allele (Fig. 6B–C′). Mutants retaining both wild-type copies of *gata5* but harboring homozygous mutations for both *gata4* and *gata6* are capable of generating cardiomyocytes and a relatively short heart tube (as shown above with embryos derived from *gata4^+/−^; gata6^+/−^* parents), underlining the importance of *gata5* in zebrafish cardiac specification (Fig. 6B). However, the additional loss of a single copy of *gata5* almost completely abolished the formation of cardiac tissue in *gata4^−/−^; gata5^+/−^; gata6^−/−^* embryos, even though *gata5* heterozygous embryos (that are wild type for *gata4/6*) are normal (Fig. 6B′). Similarly, *gata4^−/−^; gata5^−/−^* embryos retain substantial myocardial tissue. However, the additional loss of one copy of *gata6* in *gata4^−/−^; gata5^−/−^; gata6^+/−^* offspring results in a nearly heartless phenotype (Fig. 6C,C′). Interestingly, *gata4^−/−^; gata5^−/−^* embryos derived from these triple heterozygous adults display a bifid phenotype in contrast to the partially fused heart tubes observed in *gata4^−/−^; gata5^−/−^* mutants obtained from *gata4^+/−^; gata5^+/−^* parents (Fig. 4A).

### Gata6 regulates ventricular chamber morphogenesis

**Fig. 7.**
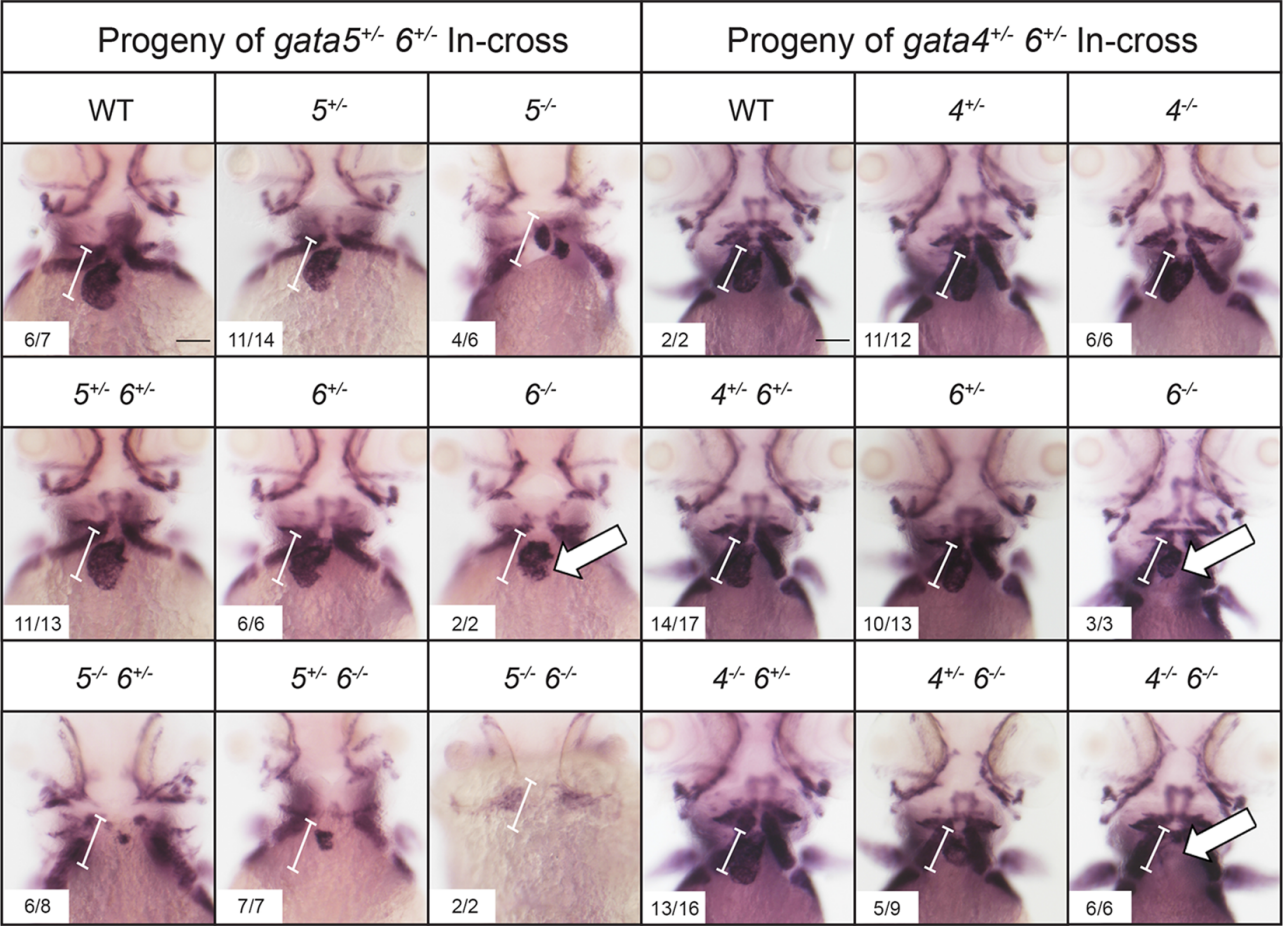
***Gata6* regulates ventricular development.** ISH was performed at 3 dpf with offspring obtained from crossing either *gata5^+/−^; gata6^+/−^* (left panels) or *gata4^+/−^; gata6^+/−^* (right panels) double heterozygous adult animals with a probe targeting the ventricular marker *vmhc*. The *gata6* homozygous mutants display smaller ventricles (white arrows) compared to the ventricles in wild-type siblings (the normal size of a wild-type ventricle is shown by the bracketed white line, which helps to indicate relative small size of the chamber in mutant embryos). The *gata4* null embryos display progressively smaller ventricles with loss of *gata6* alleles (white arrows). Representative scale bars in WT panels: 0.1 mm.

**Fig. 8.**
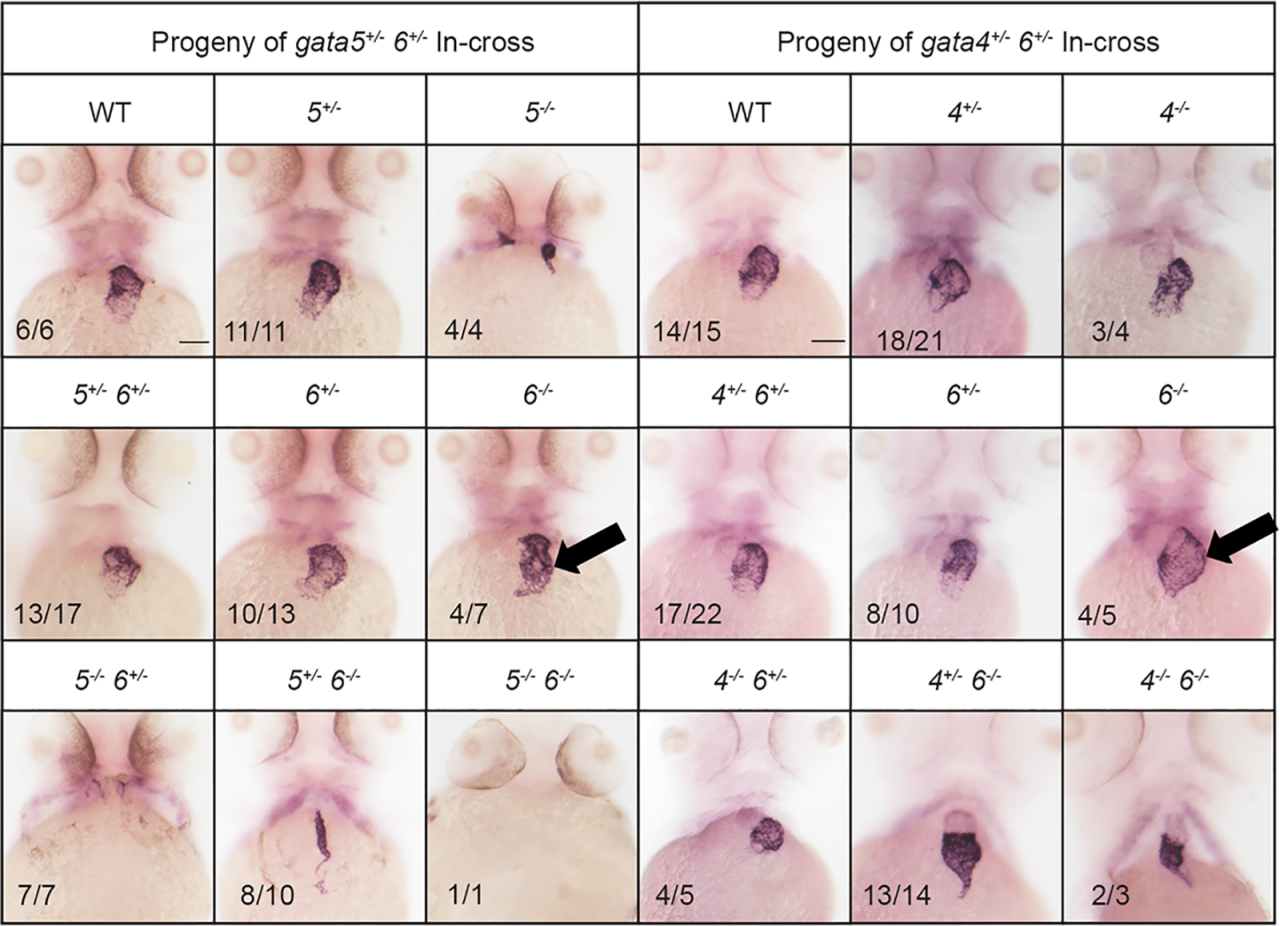
***Gata6* mutants exhibit expanded atrial chamber area.** ISH was performed at 3 dpf with offspring obtained from crossing either *gata5^+/−^; gata6^+/−^* (left panels) or *gata4^+/−^; gata6^+/−^* (right panels) double heterozygous adult animals with a probe targeting the atrial marker *amhc*. The *gata6* homozygous mutant hearts exhibit increased *amhc* staining compared to *gata4* or *gata5* homozygous mutants or wild-type siblings (black arrows). Representative scale bars in WT panels: 0.1 mm.

**Fig. 9.**
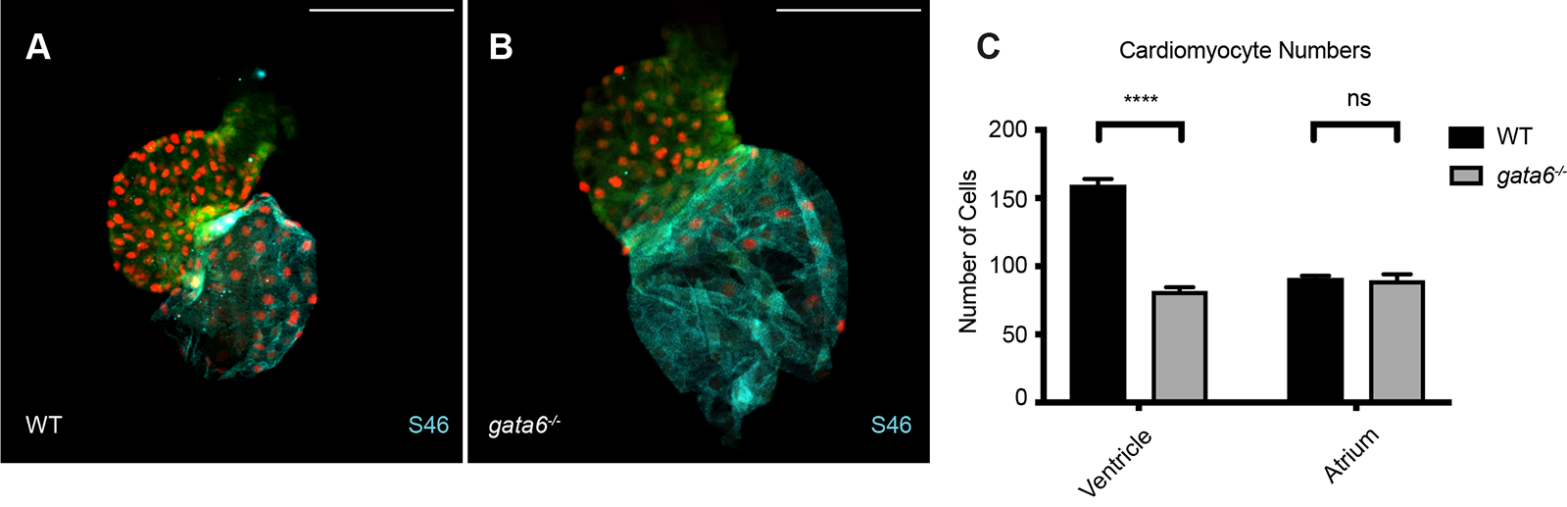
***Gata6* regulates the number of ventricular cardiomyocytes.** Shown are representative images of sibling wild-type (WT) (A) and *gata6* null hearts (B) at 2 dpf derived from crossing *Tg(myl7:gfp; myl7:dsREDnuc); gata6+/-* adults and stained with S46 antibody to distinguish the atrium (blue). Scale bars: 0.1 mm. (C) Quantification of cardiomyocytes in each chamber shows a significant loss of ventricular cardiomyocytes in *gata6* homozygous mutants (*N*=7 per genotype, *t*-test, *P*<0.0001, bars shown are s.e.m.). The number of atrial cells in the mutant remains unaffected compared to sibling controls, even though the atrial area is expanded.

To assess chamber specific phenotypes in the *gata4/5/6* mutant embryos, ISH was performed at 3 dpf to analyze both ventricular and atrial chamber formation. At 3 dpf, *gata6^−/−^* embryos obtained from crosses of *gata5^+/−^; gata6^+/−^* or *gata4^+/−^; gata6^+/−^* adults displayed small ventricles compared to heterozygous or wild-type siblings (Fig. 7, white arrows). As shown above (Figs 3 and 5) these embryos are normal for specification of cardiac progenitors, so this represents a chamber morphogenetic defect. As expected from the previous crosses, at 3 dpf *gata5/6* null embryos lacked staining for either *vmhc* or *amhc*, consistent with an earlier defect in cardiac specification. In addition, *gata4/6* double mutants exhibited smaller ventricles compared to *gata6* single mutants, with a progressive loss of ventricular tissue detected following the additional loss of *gata4* alleles in embryos with a *gata6^−/−^* background (Fig. 7, white arrows). We note that in the *gata4*^+/−^;*gata6*^−/−^ embryos the *vmhc* phenotype was not fully penetrant. Only about half the embryos showed reduced staining, with the others either showing a thin chamber (2/9) or wild-type chamber (2/9). In contrast, ISH analysis of 3 dpf embryos revealed an expansion of the atrial chamber area in *gata6^−/−^* mutants (Fig. 8, black arrows). To determine whether this expansion of atrial tissue is due to an increase in the number of atrial cells (perhaps at the expense of the ventricular cells), cardiomyocytes were counted in 2 dpf *Tg(myl7:GFP; myl7dsREDnuc)* transgenic reporter fish that were either wild type or carried *gata6^−/−^* alleles, and co-stained for the S46 atrial marker (Fig. 9A,B). The number of GFP(+) atrial cells co-staining with an S46 antibody was not different in mutants compared to siblings. In contrast, the number of GFP(+) S46(−) cells in the ventricular chamber was decreased by approximately 50% in *gata6* null hearts (Fig. 9C). Therefore, the atrial phenotype is not due to a defect in cell number but likely due to hemodynamic insufficiency-induced morphology changes, while the ventricular defect is due to loss of cardiac cell numbers in the *gata6* mutants.

**Fig. 10.**
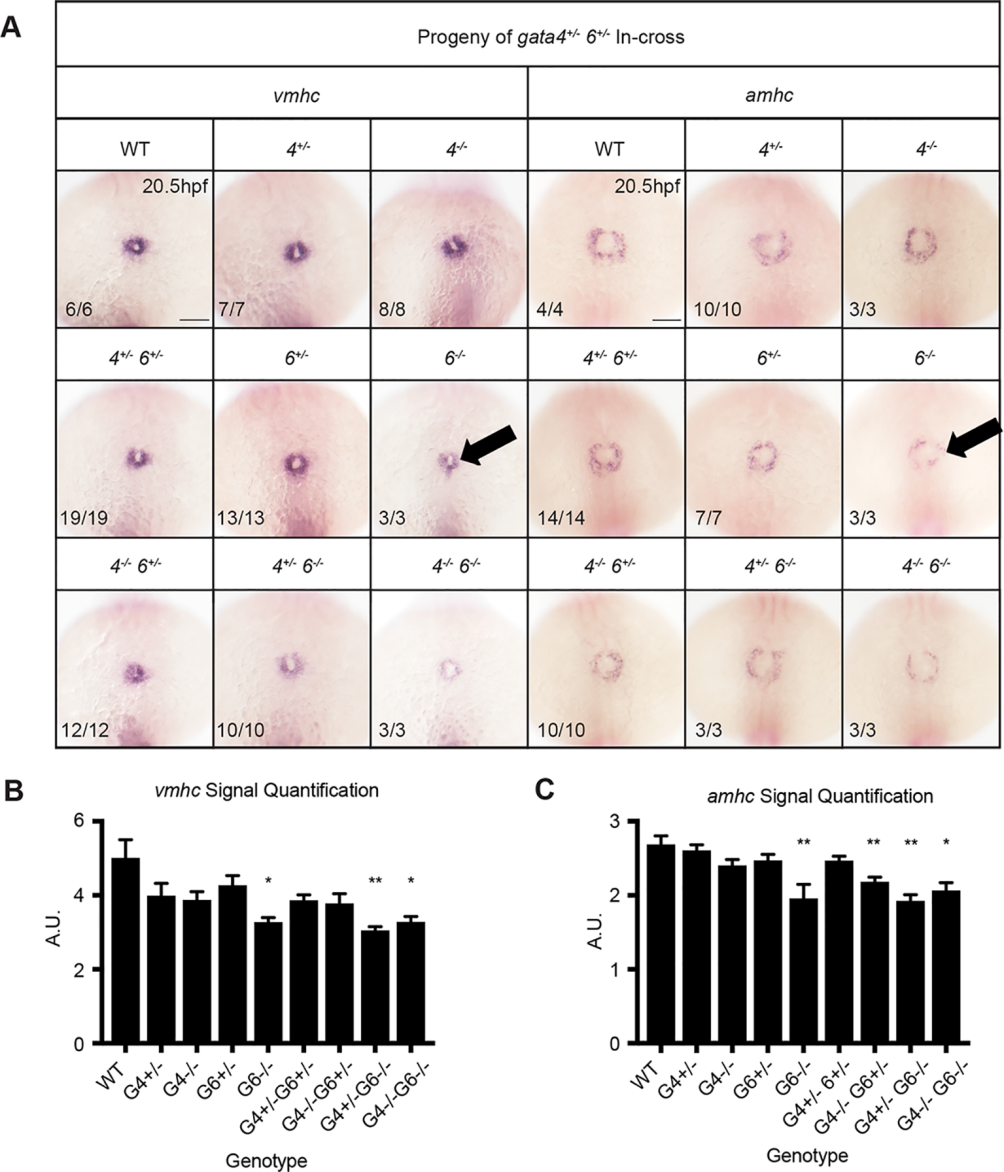
**First heart field differentiation is reduced in *gata6* mutants.** ISH was performed using embryos of the indicated genotypes at 20.5 hpf using probes targeting *vmhc* or *amhc*. (A) Ventricular and atrial cardiomyocyte differentiation is decreased in *gata6* homozygous mutant embryos (arrows). Representative scale bars in WT panels: 0.1 mm. (B,C) Quantification of *vmhc* and *amhc* staining shown in A supports a significant loss of differentiation in *gata6* null embryos. Error bars are s.e.m. *P<0.05, **P<0.01.

**Fig. 11.**
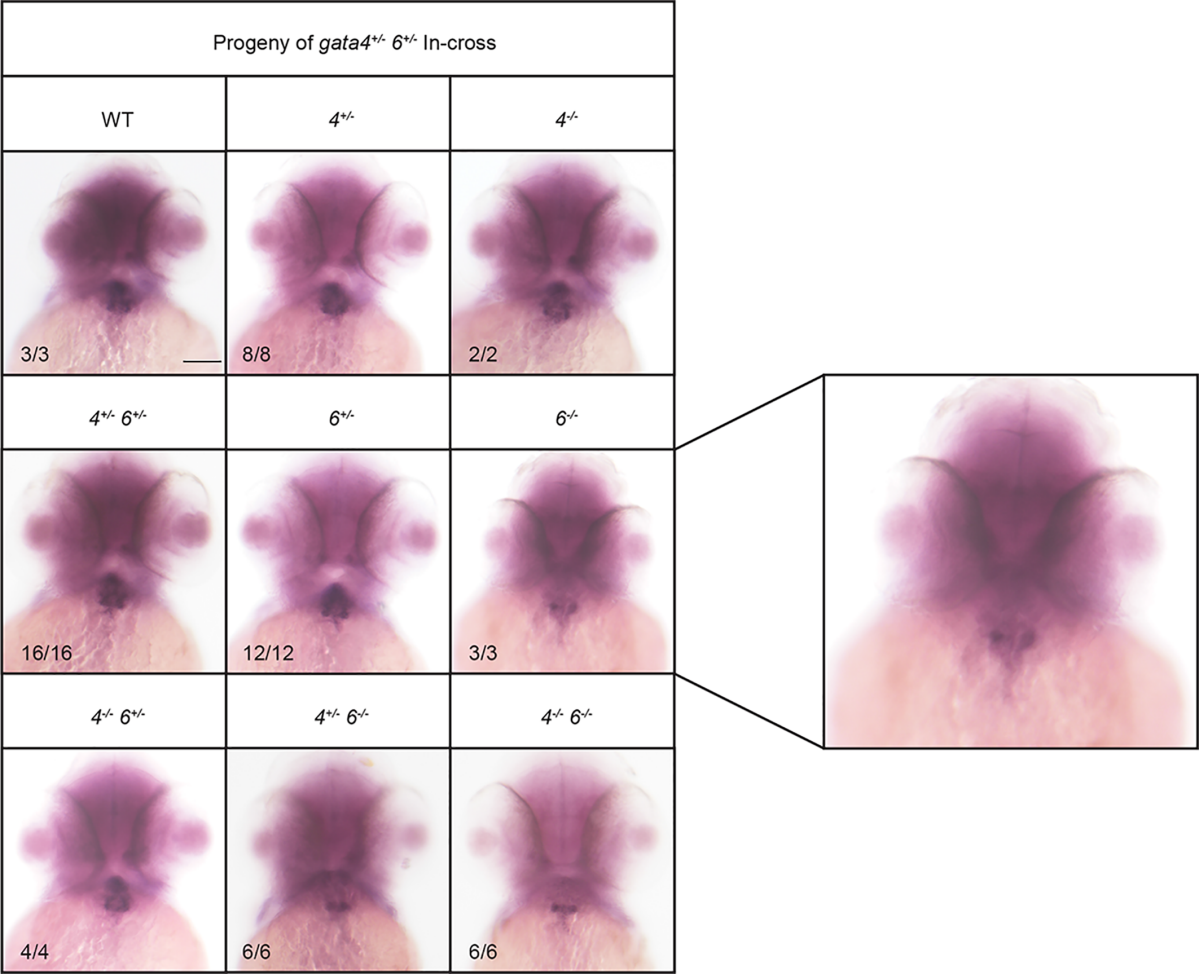
**Loss of *gata6* impairs second heart field development.** ISH was performed using embryos with the indicated genotypes at 2 dpf using a probe targeting second heart field marker *ltbp3*. The *gata6* null embryos express lower levels of *ltbp3* compared to *gata4* null and wild-type (WT) siblings. Representative scale bar in WT panel: 0.1 mm. Panel on the right is a higher magnification view of representative *gata6* mutants.

Formation of the embryonic heart including extension of the ventricular chamber requires recruitment of early and late differentiating cardiomyocytes, referred to as derived from the first and second heart fields (FHF and SHF), respectively. Analysis during formation of the FHF at 20.5 hpf revealed a decrease in *vmhc* expression in *gata6^−/−^* embryos (Fig. 10, left panels), indicating compromised differentiation of FHF derived cardiomyocytes. Note that this occurs in *gata6* mutant embryos that have normal expression of *nkx2.5*, suggesting that the FHF ventricular defect occurs subsequent to cardiac progenitor specification. To assess whether this FHF phenotype results from delayed differentiation, ISH was performed again at 26 hpf after heart tube elongation has already commenced (Fig. S5). *Vmhc* staining at this time point was clearly reduced in *gata6* mutants, confirming a chamber specific role for *gata6* in regulating ventricular cardiomyocyte differentiation in the FHF. We also examined whether *gata6^−/−^* hearts exhibit a reduction in late differentiating cardiomyocytes by analyzing the expression levels for the SHF marker *ltbp3* (Zhou et al., 2011)*.* At 2 dpf, *gata6* null embryos showed a clear reduction in *ltbp3* expression in the heart, suggesting impaired development of the SHF derivatives (Fig. 11). Decreased expression of *ltbp3* was observed only in mutants homozygous for loss of *gata6.* The additional loss of *gata4* alleles in embryos harboring a *gata6* null background did not exacerbate the phenotype, suggesting the defect is primarily due to loss of *gata6* function. Taken together, the data are consistent with the loss of *gata6* resulting in impaired ventricular cardiomyocyte development from both the FHF and SHF.

## DISCUSSION

The observation that *gata4^−/−^* mutant embryos survive early development is surprising considering previously published morphant phenotypes and the embryonic lethal phenotype described in *Gata4* mutant mice. However, the mutant fish are not entirely normal. Their early development is delayed and they show a relative upregulation of *gata6* mRNA suggesting that *gata6* might be able to compensate for the early loss of *gata4*. This conclusion would be consistent with other examples of GATA sister-gene compensation observed in murine models (Molkentin et al., 1997; Singh et al., 2010). Furthermore, the severe cardiomyopathy observed in *gata4^−/−^* adult fish indicates that compensation is limited and that *gata4* is required to maintain cardiac homeostasis later in adulthood. Why loss of *gata4* causes an age-dependent cardiomyopathy remains to be determined. The possibility that the targeted mutations are not null is remote. All the evidence in the literature indicates that for GATA factors, loss of the C-terminal zinc-finger eliminates DNA-binding and transcriptional activity. However, recent studies have shown that degradation of mutant mRNA can trigger the upregulation of related genes, which does not occur for deletion alleles that do not produce any RNA (Tuladhar et al., 2019). Therefore, it is possible that the mutant *gata4* alleles stimulate genetic compensation by *gata6* during embryogenesis.

Zebrafish genetics provides strong evidence that overall levels of Gata4/5/6 are critical during early cardiogenesis rather than unique specific functions for each gene, in a much more rigorous manner than can be obtained using morphant models. We found that embryos lacking functional alleles for two of the three cardiac GATA factors (*gata4/5* or *gata4/6*) exhibit a severe loss of cardiac tissue when a single allele is lost for the third factor. Therefore, expression of only one wild-type copy of either *gata5* or *gata6* in the absence of other GATA factors reduces the total level below a threshold necessary for the development of myocardial cells. Apparently, even both alleles of *gata4* are insufficient to achieve this threshold in the absence of *gata5/6*.

Two interesting observations were made for which we do not yet have mechanistic explanations and for which we can currently only speculate. First, we found a different cardiac phenotype in the *gata4*^−/−^;*gata5*^−/−^ mutants obtained from a *gata4*^+/−^×*gata5*^+/−^ cross (Fig. 4A) compared to those obtained from the triple heterozygous cross (Fig. 6C). The *gata4* null fish may express higher levels of *gata6* (based on our single mutant qPCR data). This would presumably not be the case for the triple heterozygous fish. Second, we do not know why in the former case the loss of *gata4* rescues the *bifid* phenotype. However, we can imagine that loss of *gata4* alleles impacts expression of *gata6* or other gene(s) that interact genetically with *gata5* in the endoderm (as *cardia bifida* in *gata5* mutants is known to be an endoderm defect impacting cardiac progenitor migration).

Here we defined a previously uncharacterized function for *gata6* in chamber morphogenesis, as loss of both alleles results in a hypocellular ventricle. While the *gata4* mutant embryo does not have a strong ventricle phenotype, the absence of *vmhc* expression in *gata4^−/−^; gata6^−/−^* mutant hearts indicates that *gata4* and *gata6* are functionally redundant in ventricular development. This redundancy may at some level reflect conserved functions between the fish and mouse in generating ventricular cardiomyocytes as *Gata4/6* double knockout mice exhibit acardia (Zhao et al., 2008). However, since the *Gata4/6* double knockout mouse may be more similar to the *gata5/6* double mutant fish (heartless), it may also reflect species-specific differences for generating threshold levels necessary for cardiac progenitor specification. At 2 dpf, *gata6* null hearts had atrial cardiomyocyte numbers comparable to wild-type siblings, ruling out the possibility that an increase in atrial cells developed at the expense of ventricular differentiation. The enlarged atrial chamber is therefore likely a secondary effect of a dysfunctional ventricle or defects in valve morphogenesis or hemodynamics. The observation that *nkx2.5* staining was unaffected in *gata6* mutants also suggests that first heart field progenitor specification occurs normally and *gata6* primarily regulates the differentiation of ventricular cardiomyocytes. Taken together, these findings demonstrate a specific role for *gata6* in directing development of the ventricular chamber.

In this study, we generated and analyzed single, double and triple *gata4/5/6* mutant zebrafish strains to provide a comprehensive overview regarding the effects of single and combinatorial mutations on cardiogenesis (summarized in Table 1). The analyses of these mutants show that they phenocopy some developmental features previously described in morphants, but the genetic analysis has clear advantages especially for the ability to combine alleles, providing rigorous titration of *gata4/5/6* dose and avoiding off-targeting or toxicity that can limit the morphant model. The lack of reliance on GATA-dependent extra-embryonic endoderm, external development of the fish embryo, and its ability to withstand severe organ failure during early development suggest these mutant lines provide an especially attractive model for investigating the function of these genes in other aspects of cardiovascular development or other GATA-dependent organ systems, such as those derived from the gut.

**Table 1.**
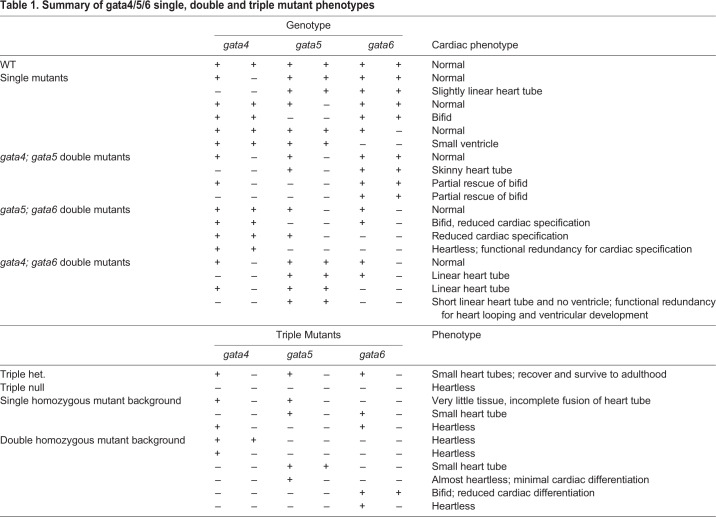
**Summary of gata4/5/6 single, double and triple mutant phenotypes**

## MATERIALS AND METHODS

### Generation of mutants

TALENs were designed using the Cornell TAL Effector Nucleotide Targeter 2.0 website (Doyle et al., 2012) and plasmids encoding specific TALENs were generated with the TALEN Golden Gate 2.0 kit (Addgene) as previously described (Cermak et al., 2011). Generation of correct sequences was confirmed through digestion with BspEI. Resulting vectors were linearized with SmaI, and mRNA encoding TALENs was synthesized with the mMessage mMachine kit (Thermo Fisher Scientific). The mRNA was quantified with a NanoDrop and analyzed by electrophoresis through a 1% agarose gel with ethidium bromide in order to verify that there was no degradation. 100–400 pg of mRNA was injected into zebrafish embryos at the 1–2 cell stage.

Guide RNAs (gRNAs) for CRISPR/Cas9 mediated mutagenesis were designed with the CHOPCHOP website (Montague et al., 2014) and ordered from IDT as double stranded oligo cassettes with a T7 promoter at the 5′ end. The gRNA was generated with the MEGAshortscript kit (Thermo Fisher Scientific) and purified through ammonium acetate precipitation as per the manufacturer's instructions. The mRNA was quantified with a NanoDrop and analyzed by electrophoresis through a 1% agarose gel with ethidium bromide in order to verify that there was no degradation. 250 ng/µl gRNA was combined with 500 ng/µl recombinant Cas9 protein (PNA BIO) and 0.2 M KCl, and incubated at room temperature for 15 min to allow RNA-protein complexes to form. 1 nl of the gRNA-Cas9 complex solution was injected into embryos at the 1–2 cell stage.

Putative F0 founder fish were raised to adulthood and outcrossed to wild-type fish (AB/Tub hybrids). Several samples of genomic DNA from each clutch (typically two to three samples from five to ten embryos) were analyzed for mutant alleles by T7 endonuclease assays following PCR, essentially as described (Zhu et al., 2014). When mutant alleles were detected, the remaining embryos were raised to adulthood, and F1 heterozygous fish were identified by genotyping similarly from fin-clips (Draper et al., 2007). PCR products were also cloned into a TOPO vector (Invitrogen) and sequenced to define each mutant allele (indicated in Table S2).

Wild-type, heterozygous, and homozygous mutant fish from each line were genotyped using a PCR assay utilizing two forward primers and one reverse primer (R) in the same reaction (Table S3). An outside forward primer (F1) was designed to bind a section of gDNA sequence upstream of the *gata* mutation while an internal forward primer (F2) was designed to anneal to the sequence deleted in the mutant allele. Homozygous mutants were identified by the larger amplicon produced by the pairing of the F1-R primers. Wild-type fish were identified by the shorter amplicon produced by the pairing of the F2-R primers, which competes the pairing of the F1-R primer set. Heterozygous animals were identified based on the production of both the F1-R and F2-R amplicons.

### Wholemount ISH

ISH was performed as described previously (Holtzinger and Evans, 2007). Briefly, embryos were fixed in 4% paraformaldehyde, dehydrated in methanol, and stored at −20°C overnight. Embryos older than 24 hpf were treated with 10 µg/ml proteinase K (30 hpf embryos: 5′; 2 dpf embryos: 10′; 3 dpf embryos: 15′). Hybridization was performed using digoxigenin-labeled RNA anti-sense probes at 70°C. *Amhc*, *vmhc*, *myl7* and *nkx2.5* probes were described previously (Holtzinger and Evans, 2007). The probe for *ltbp3* was generated using primers (F: 5′ TTAGGATCCACAACACCACTCTCATCGGT 3′; R: 5′ ATTACTCGAGCAGTTGCTTCCCCATGCTTT 3′) designed with BamHI and XhoI restriction sites. A 946 bp region of the coding sequence was amplified from cDNA collected from 2 dpf wild-type embryos, purified using a QIAquick PCR Purification Kit (Qiagen), and cloned into the pCS2+ vector. Sense and anti-sense probes were *in vitro* transcribed using the mMESSAGE mMACHINE kit (Thermo Fisher Scientific) and purified using Microspin G-50 columns (GE Healthcare).

### ISH quantification

ISH experiments were performed as described above using 15-somite stage embryos and digoxigenin-labeled RNA anti-sense probes targeting *nkx2.5*, or 20.5 hpf embryos and probes targeting either *vmhc* or *amhc*. Custom MATLAB codes were created and used to semi-quantitatively measure the staining from each probe. In each case, signal quantification was normalized to the background staining of the whole embryo. Due to the variable amount of background staining that can occur during the ISH staining process, the calculated normalized staining levels can vary somewhat in each assay. As a result, we only compared staining levels across embryos obtained from the same experimental cohort. Average normalized signals for each genotype were then compared to the average signal for wild-type samples using a one-way ANOVA test on GraphPad Prism (version 8.2.1 for MacOS, GraphPad Software, La Jolla, CA, USA, www.graphpad.com).

### Protein collection

For each of three replicates, five hearts (bulbus arteriosus and ventricle) were dissected from either wild-type or *gata4^−/−^* adult zebrafish. Hearts were placed in 800 µl cold lysis buffer (20 mM Tris pH7.5, 150 mM NaCl, 50 mM NaF, 1% NP-40) containing 1:100 protease/phosphatase inhibitor cocktail (Cell Signaling Technology), processed using a BioSpec Tissue Tearor Homogenizer in 10 s intervals on ice for a total of 2 min, and then incubated at 4°C for 1 h. Samples were then concentrated using acetone protein precipitation. Samples were first vortexed in a 4× volume of cold acetone and incubated for 1 h at −20°C. Each sample was centrifuged and pellets were resuspended in 40 µl 1× sample buffer containing NuPAGE LDS sample buffer and reducing agent (Invitrogen).

### Western blotting

Primary antibodies specific to Gata4 were generated by and purchased from Yenzym Antibodies, LLC. Rabbits were immunized with a combination of Gata4-derived peptides (residues 70-80: NSSTGHHHSPVSR and residues 333-347: GSPSGSSSSKSEVWN). Protein samples were boiled at 95°C in 1× sample buffer and run on a NuPAGE 1.5 mm 10% Bis-Tris gel (Invitrogen). Following gel electrophoresis, semi-dry transfers were performed using iBlot PVDF Transfer Stacks (Invitrogen). Membranes were blocked (3% BSA, 1× TBS, 0.1% Tween-20) at room temperature then incubated in a primary antibody for Gata4 (1:250) overnight at 4°C and HRP-conjugated secondary antibody (1:2000, Bio-Rad) for 1 h at room temperature. Membranes were then treated with Pierce ECL Western Blotting Substrate (Thermo Fisher Scientific). Following gel electrophoresis, Coomassie staining was performed using SimplyBlue SafeStain (Invitrogen).

### Cell counts

*Tg(myl7:GFP; myl7;dsREDnuc)* wild-type and *Tg(myl7:GFP; myl7;dsREDnuc); gata6^−/−^* embryos were fixed at 2 dpf in 4% paraformaldehyde and incubated in blocking buffer (0.2% saponin, 1× PBS, 10% lamb serum) for 1 h at room temperature. Samples were incubated in primary antibodies for S46 (1:15, Developmental Studies Hybridoma Bank) or dsRED (1:1000, Clontech) overnight at 4°C and secondary antibodies (1:1000, Life Technologies) for 2 h at room temperature. Hearts were dissected using fine forceps and mounted in 1% low melt agarose before being imaged using a Zeiss LSM800 laser scanning confocal microscope. Z-stacks were analyzed in Fiji and dsRED positive nuclei were counted using the Cell Counter plugin (Schindelin et al., 2012). Cell numbers were compared using a *t*-test performed using GraphPad Prism (version 8.2.1 for MacOS, GraphPad Software, La Jolla, CA, USA, www.graphpad.com).

## Supplementary Material

Supplementary information
