# Supplementary material for: Specificity, redundancy and dosage thresholds among gata4/5/6 genes during zebrafish cardiogenesis
Source: Biol Open. 2020 Jun 24;9(6):bio053611. doi: 10.1242/bio.053611 (PMC7327998; doi:10.1242/bio.053611)
Supplement: Supplementary information [file biolopen-9-053611-s1.pdf]

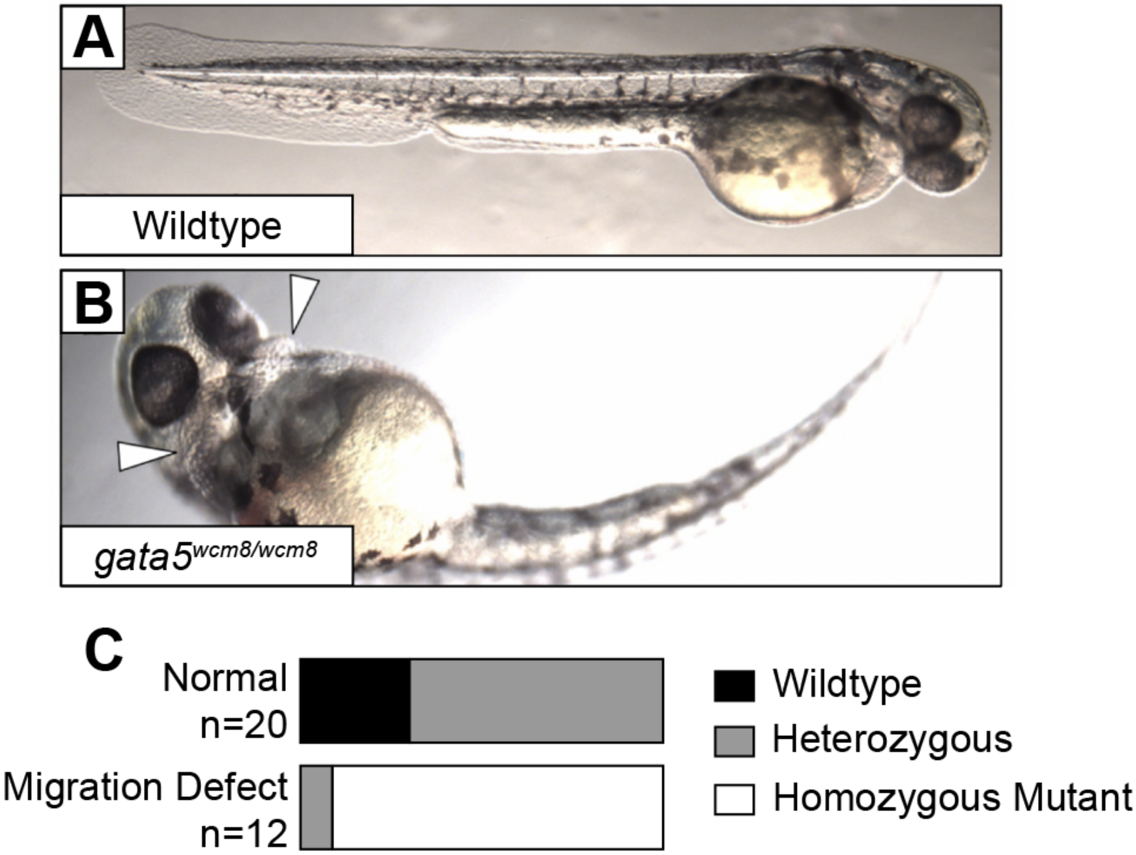

Supplemental Figure S1. *Gata5* mutants exhibit *cardia bifida* by 2 dpf. (A-B) *Gata5* homozygous mutant embryos exhibit *cardia bifida* (white arrowheads) and pericardial edema compared to wildtype embryos. (C) The *cardia bifida* phenotype is 100% penetrant among *gata5* homozygous mutant offspring collected from a cross of *gata5*$^{+/-}$ adults.

Biology Open • Supplementary information

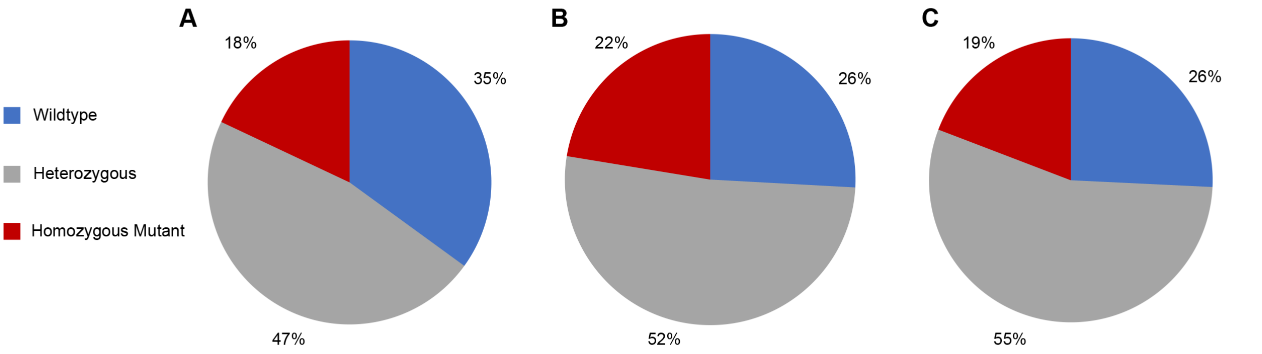

Supplemental Figure S2. *Gata4* mutants are not recovered in expected ratios as larvae or adults. (A) Percentage of wildtype, heterozygous, or homozygous mutant 3 dpf offspring obtained from crosses of *gata4*$^{wcm5/+}$ and *gata4*$^{wcm6+/-}$ adults (N=513). (B-C) Percentage of each genotype found in adult offspring from crosses of *gata4*$^{wcm5/+}$ (B, N=58) and *gata4*$^{wcm6+/-}$ (C, N=198) parents.

Biology Open • Supplementary information

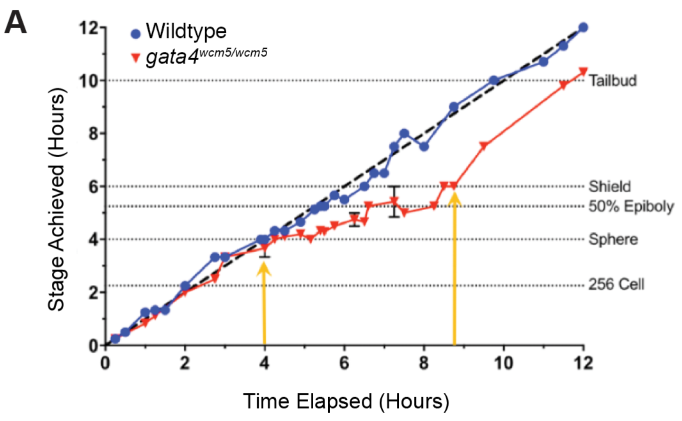

Supplemental Figure S3. *Gata4* homozygous mutants undergo delayed gastrulation. (A) Schematic visualizing the length of time wildtype and mutant embryos take to reach each developmental stage. Points are mean time +/- S.D. and yellow lines mark the period of delay. (B) Quantification of time taken for embryos to reach specific stages of development (n=3 batches).

A

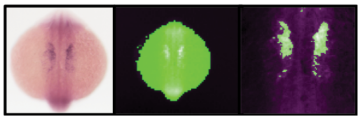

D

A. Signal Area (a.u.)	B. Total Signal Intensity (a.u.)	C. Mean Signal Intensity (B/A) (a.u.)	D. Mean Background Intensity (Whole Embryo) (a.u.)	E. Signal Relative to Whole Embryo (C/D) (a.u.)
27577.25	4359363	158.0782	98.54654	1.604097

B

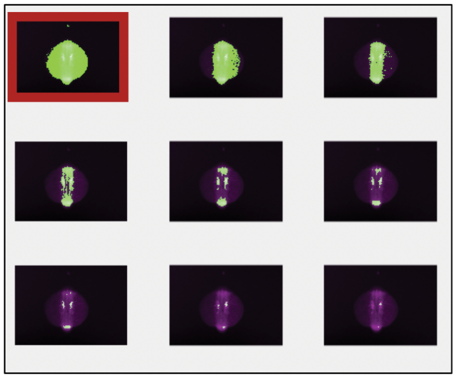

C

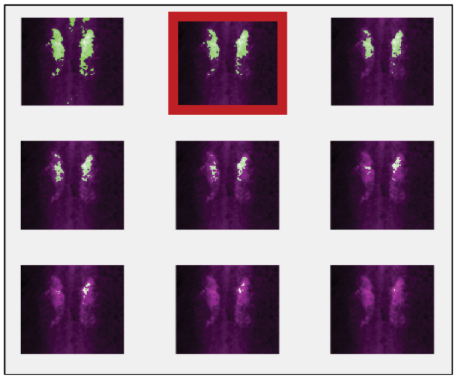

Supplemental Figure S4. MATLAB quantification of ISH staining. Quantification was performed using a custom MATLAB code with probe staining normalized to background staining of the whole embryo. (A) Representative example of original ISH image (left) compared to MATLAB quantified background staining (middle) and probe staining (right). (B-C) Examples of threshold levels set to quantify background and probe signal. (D) Example of output data with results from column E used to compare each mutant to wildtype.

Biology Open • Supplementary information

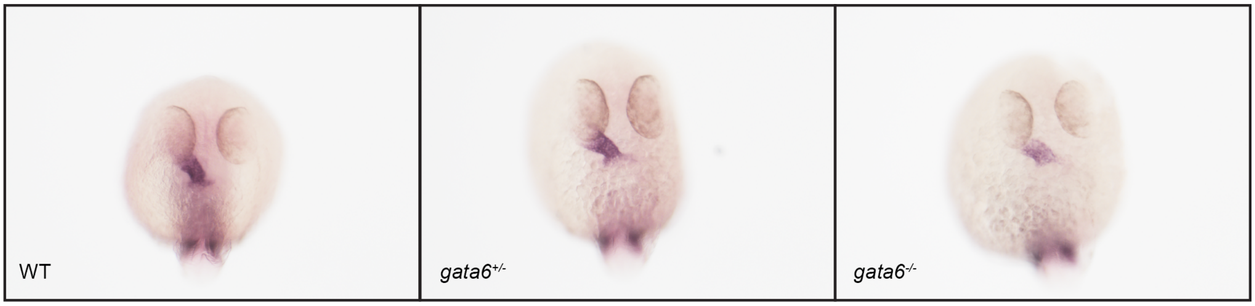

Supplemental Figure S5. **Reduced ventricular differentiation in *gata6* mutant embryos.** ISH performed at 26 hpf shows reduced expression of *vmhc* in *gata6*$^{-/-}$ embryos compared to wildtype (WT) or heterozygous siblings.

Biology Open • Supplementary information

CRISPR SEQUENCE		
GENE	gRNA TARGETED SEQUENCE **(PAM)**	
gata5	5'- GGGCGCGAGTGTGTGAACTG**(CGG)**-3'	
**TALEN SEQUENCES**		
GENE	TARGETED SEQUENCE	RVD SEQUENCES
gata4	5'-GCGTCCAGGCGGGTGG-3'	NH HD NH NG HD HD NI NH NH HD NH NH NH NG NH NH
	5'-GTCAGACTACCACAAC-3'	NH NG NG NH NG NH NH NG NI NH NG HD NG NH NI HD
gata6	5'-CTTCCTCCCGGCGGATCGGA-3'	HD NG NG HD HD NG HD HD HD NH NH HD NH NH NI NG HD NH NH
	5'-TGTCAGACGAGCACCACAAC-3'	NH NG NG NH NG NH NH NG NH HD NG HD NH NG HD NG NH NI HD

Supplemental Table 1. CRISPR and TALEN sequences.

Biology Open • Supplementary information

ALLELE	GENE	DNA SEQUENCE
WT	*gata4*	GGGTGGGTTTATCCTGCACAAACTGTCAGACT
wcm5	*gata4*	GGGTGGGT------------------CAGACT
wcm6	*gata4*	GGGTGGGTTTATAAACTGGGTTTACAAACTGT
WT	*gata5*	CCGTGTGAAGGGCGCGAGTGTGTGAACTGCGGCTCAATCTCAACGC
wcm8	*gata5*	CCGTGTGAAGGGCGCGAGTGTG----------CTCAATCTCAACGC
wcm9	*gata5*	CCGTGTGA-----------------CCTGCGGCTCAATCTCAACGC
wcm10	*gata5*	CCGTGTGAAGGGCGCGAGTGTGTG------------------ACGC
wcm11	*gata5*	CCGTGTGAAGGGCGCGAGTGTGT-----------------CAACGC
WT	*gata6*	GGACTGTCATGCGCAAACTGTCAGACGAG
wcm7	*gata6*	GGACTGTCA--------------GACGAG

Supplemental Table 2. ***Gata4/5/6*** **mutant alleles and corresponding DNA sequences altered by TALEN (*gata4* and *gata6*) or CRISPR (*gata5)* mediated deletion of the GATA zinc finger DNA-binding domain.**

Biology Open • Supplementary information

GENOTYPING PRIMERS		
TE #	GENE	PRIMER SEQUENCE (5' to 3')
6311 (Forward 1)	*gata4*	TTGCAGTGATTATTTATGCACATT
5995 (Forward 2)	*gata4*	GGTGGGTTTATCCTGCACA
6712 (Reverse)	*gata4*	CAGCAGACAGGACACTCACC
6074 (Forward 1)	*gata5*	GGACTGTCATGCGCAAACT
6346 (Forward 2)	*gata5*	AAATATTTGTGGAATTGATGATCC
6075 (Reverse)	*gata5*	TGTTCAAAGCAAAGGGAGAG
6313 (Forward 1)	*gata6*	CGCGAGTGTGTGAACTGC
5994 (Forward 2)	*gata6*	CAAGTCGCACCTTGAAAACA
6711 (Reverse)	*gata6*	TGGCTAGGTTTAAACAGGACAA

Supplemental Table 3. Primer sequences for genotyping *gata4/5/6* mutant fish.
